# Sleep Timing and Risk of Dementia Among the Chinese Elderly in an Urban Community: The Shanghai Aging Study

**DOI:** 10.3389/fneur.2021.629507

**Published:** 2021-04-29

**Authors:** Xiantao Li, Ding Ding, Qianhua Zhao, Wanqing Wu, Zhenxu Xiao, Jianfeng Luo, Kristine Yaffe, Yue Leng

**Affiliations:** ^1^Department of Critical Care Medicine, Huashan Hospital, Fudan University, Shanghai, China; ^2^Institute of Neurology, Huashan Hospital, Fudan University, Shanghai, China; ^3^Department of Biostatistics, School of Public Health, Fudan University, Shanghai, China; ^4^Departments of Psychiatry and Behavioral Sciences, Neurology, and Epidemiology, University of California, San Francisco, San Francisco, CA, United States; ^5^Department of Neurology, Memory and Aging Center, Global Brain Health Institute, University of California, San Francisco, San Francisco, CA, United States; ^6^Department of Psychiatry and Behavioral Sciences, University of California, San Francisco, San Francisco, CA, United States

**Keywords:** sleep, dementia, epidemiological analysis, longitudinal, low- and lower-middle-income countries

## Abstract

**Background:** Growing evidence has suggested a link between poor sleep quality and increased risk of dementia. However, little is known about the association between sleep timing, an important behavior marker of circadian rhythms, and dementia risk in older adults, and whether this is independent of sleep duration or quality.

**Methods:** We included data from 1,051 community-dwelling older men and women (aged≥ 60y) without dementia from the Shanghai Aging Study. At baseline, participants reported sleep timing, duration, and quality using the Chinese version of the Pittsburgh Sleep Quality Index (CPSQI). Dementia diagnosis over the following 7.3 years was determined by neurologists using DSM-IV criteria. We used Cox proportional hazards models to examine the association between bedtime (before 9 p.m., after 11 p.m. vs. 9–11 p.m.), rise time (before 6 a.m., after 8 a.m. vs. 6–8 a.m.), and risk of dementia.

**Results:** A total of 238 (22.8%), 675 (64.5%), and 133 (12.7%) participants reported going to bed before 9 p.m., between 9 and 11 p.m., and after 11 p.m., respectively, while 272 (26%), 626 (59.9%), and 148 (14.2%) reported getting up before 6 a.m., between 6 and 8 a.m., and after 8 a.m., respectively. Participants who reported going to bed earlier had a lower education level, were less likely to be smokers, more likely to have hypertension or diabetes, and had longer sleep duration but poorer sleep quality compared to those who reported a later bedtime. We found 47 incidents of dementia among 584 participants followed up over an average of 7.3 years. After adjustment for demographics, education, income, body mass index, depressive symptoms, smoking, alcohol use, physical activity, comorbidities, APOE4 genotype, and baseline MMSE, those with a bedtime of before 9 p.m. were two times more likely to develop dementia [hazard ratio (HR)=2.16 (95%CI: 1.06–4.40)], compared to those going to bed between 9 and 11 p.m. Later bedtime (i.e., after 11 p.m.) showed the opposite but had a non-significant association with dementia risk (HR=0.15, 95%CI: 0.02–1.29). We did not find an association for rise time and risk of dementia.

**Conclusion:** Earlier sleep timing in older adults without dementia was associated with an increased risk of dementia. Future studies should examine the underlying mechanisms of this association and explore the usefulness of sleep timing as a preclinical marker for dementia.

## Introduction

Sleep patterns change markedly with age, including altered sleep timing and duration, poor sleep quality, and increased sleep disturbances (1). These sleep changes are often more severe among those with neurodegenerative diseases including severe cognitive impairment, and have also been associated with increased risk of developing dementia. Growing evidence suggests a relationship between circadian rhythm disruption and risk of neurodegenerative diseases (2). Prospective studies reported a 1.5-2 fold increase in dementia risk associated with lower sleep efficiency, longer sleep latency, sleep-disordered breathing, or long daytime napping (3–5). However, the association between sleep timing, an important behavior marker of circadian rhythms, and risk of dementia is poorly understood. On the other hand, the impact of sleep duration or quality on such an association also needs careful consideration.

Compared to western populations, the Chinese elderly are much more likely to sleep and rise earlier, and take a nap in the afternoon. Factors owing to the genetics, culture, environment, or lifestyle of the Chinese population are also different from the western population. Through the prospective phase of the Shanghai Aging Study, the current study aimed to determine the longitudinal association between reported sleep timing and risk of dementia among older Chinese adults, and to study whether this association is independent of sleep duration or quality.

## Methods

### Study Design and Participants

During 2010–2011, community residents aged 60 years or older were consecutively enrolled based on a government-maintained residents list of the Jingansi community in central Shanghai. Participants were excluded if they: (1) had severe mental delay or schizophrenia; (2) had difficulties of vision, hearing, or speaking, and (3) were not capable of accomplishing a neuropsychological evaluation. A detailed description of the design and procedure of the Shanghai Aging Study has been published elsewhere (6).

This study was approved by the Medical Ethics Committee of Huashan Hospital, Fudan University, Shanghai, China. A written informed consent was obtained from all of the participants and/or their legal guardian.

### Participant Characteristics

Study participants underwent a clinical interview either at Huashan Hospital or at their homes. At baseline, demographic characteristics were collected via an interviewer-administered questionnaire including age, sex, education, income, cigarette smoking, alcohol consumption, and physical activity. Cigarette smoking was defined as a person who had smoked daily within the past month. Alcohol consumption was defined as a person who had had at least one episode of alcohol drinking weekly during the past year. Medical histories such as hypertension, diabetes, and heart diseases were self-reported and further confirmed from medical records. Body mass index (BMI) was calculated as weight in kilograms (kg) divided by height in meters (m) squared. High depressive symptoms were determined to be present if the Center for Epidemiologic Studies Depression Scale (CESD) score was ≥16 (7).

DNA was extracted from blood or saliva samples from each participant to conduct Apolipoprotein E (APOE) genotyping by the Taqman SNP method (8). Presence of at least one ε4 allele was classified as APOE ε4 positive.

### Measurement of Sleep Quality

At baseline, participants reported sleep quality over a one-month time interval through the Chinese version of the Pittsburgh Sleep Quality Index (CPSQI), which contains seven “component” scores: sleep quality, latency, duration, habitual sleep efficiency, sleep disturbances, use of sleeping medication, and daytime dysfunction (9, 10). A global score of subjective sleep quality (range 0–21) was then determined by the sum of the seven component scores with the higher scores representing poorer subjective sleep quality (10). Participants were asked their bedtime, time of falling asleep, rise time, and sleep duration. Bedtime was categorized to “before 9 p.m., after 11 p.m., and 9–11 p.m.”; and rise time was categorized to “before 6 a.m., after 8 a.m., and 6–8 a.m.”

### Neurological, Neuropsychological Assessments, and Diagnosis

At baseline, neurologists examined each participant for their motor responses and reflexes. Each participant was administered a battery of neuropsychological tests for global cognition, executive function, spatial construction function, memory, language, and attention. The battery contained (1) the Mini-Mental State Examination; (2) Conflicting Instructions Task (Go/No Go Task); (3) Stick Test; (4) Modified Common Objects Sorting Test; (5) Auditory Verbal Learning Test; (6) Modified Fuld Object Memory Evaluation; (7) Trail-making tests A and B; and (8) RMB (Chinese currency) test. Participants with ≥6 years of education were given tests 1 to 5, and 7; and all others were given tests 1 to 4, 6, and 8. Normative data and more details of these tests were reported elsewhere (11). All tests were conducted in Chinese by study psychometrists. Neurologists also administered the Clinical Dementia Rating (CDR) (12) and the Lawton and Brody scale of Activity of Daily Living (ADL) (13) to elicit memory complaints and functional abilities.

A panel of neurologists, neuropsychologists, and research coordinators reviewed all the examinations for each participant at baseline and reached a consensus diagnosis for dementia using DSM-IV criteria (14). Detailed diagnostic procedures have been reported elsewhere (6).

### Follow-Up Procedure

From April 1, 2014 to December 31, 2016, dementia-free participants with complete sleep quality data at baseline were evaluated at follow-up. Cognitive function was evaluated using the same neuropsychological battery at baseline. Consensus diagnosis of incident dementia was conducted by the same panel of experts using the same diagnostic criteria as at baseline.

### Statistical Analysis

Mean with standard deviation (SD), or median (Q1, Q3), and number with frequency (%) were used to describe continuous and categorical variables, respectively. Student's *t*-test was used to analyze the differences for continuous variables, while Pearson's chi-squared test was used to analyze the differences for categorical variables. The incidence of dementia was calculated as the number of new-onset dementia cases divided by the cumulative person-years of follow-up period. Cumulative incidence of dementia were estimated with the Kaplan-Meier product-limit method and compared by the log-rank test. Cox proportional hazards models were used to estimate adjusted hazard ratios (HRs) with 95% confidence intervals (CIs) for the association between bedtime (before 9 p.m., after 11 p.m. vs. 9–11 p.m.), rise time (before 6 a.m., after 8 a.m. vs. 6–8 a.m.), and risk of dementia. Model 1 adjusted for age, sex, education. Model 2 further adjusted for cigarette smoking, alcohol consumption, hypertension, diabetes, heart disease, stroke, APOE ε4, BMI, and MMSE. In further sensitivity analysis, we additionally adjusted sleep duration and quality as covariables. Model 3 additionally adjusted for covariables including sleep duration and efficiency.

All the *p*-values and 95% CIs were estimated in a two-tailed manner. Differences were statistically significant at *p* < 0.05. Data were analyzed using SAS 9.4 (SAS Institute Inc., Cary, NC, USA).

## Results

**Figure 1:**
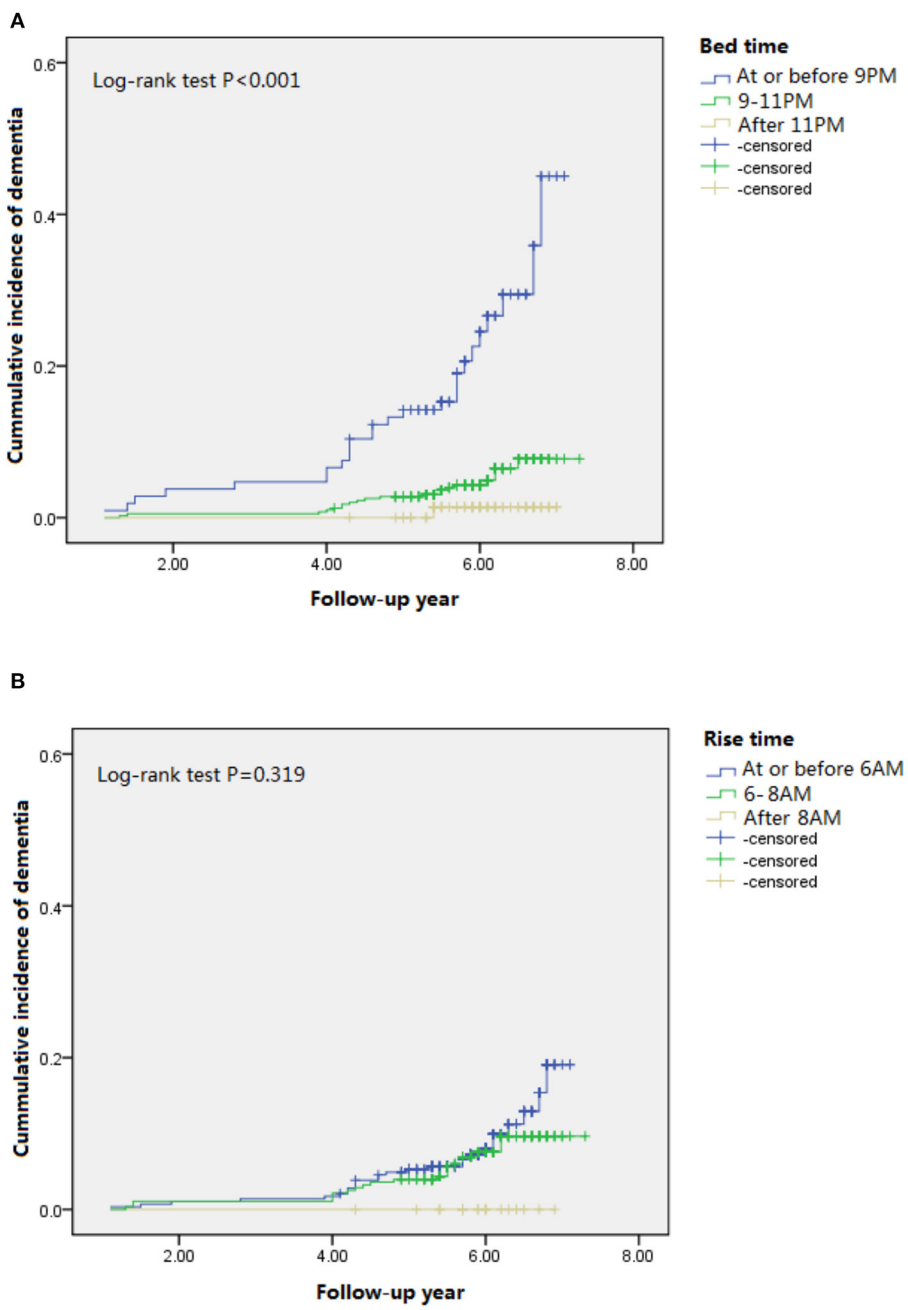
Cumulative incident plots of dementia in participants with different bedtime **(A)** and rise time **(B)** during the follow-up.

We evaluated 1,051 community-dwelling older men and women without dementia. As shown in Table 1, 238 (22.8%), 675 (64.5%), and 133 (12.7%) participants reported going to bed before 9 p.m., between 9 and 11 p.m., and after 11 p.m., respectively. A total of 272 (26%), 626 (59.9%), and 148 (14.2%) participants reported getting up before 6 a.m., between 6 and 8 a.m., and after 8 a.m., respectively. The “early birds” (who reported going to bed earlier) had a lower education level, were less likely to be smokers and drinkers, less likely to do physical activity, more likely to have hypertension or diabetes, more likely to be an APOE e4 carrier, and had lower MMSE scores at baseline and follow-up compared to the “night owls” (who reported going to bed later). Over an average of 7.3 years of follow-up, we found 47 (8.0%) incident dementia cases among 584 study participants who completed the follow-up interview. The highest dementia incidence was found in participants who reported going to bed before 9 p.m., and the lowest dementia incidence was found in those who reported going to bed after 11 p.m. (*p* < 0.001). Significant difference of the cumulative incidence of dementia was found among three groups when reporting bedtime (Figure 1A), but not among those three groups when reporting rise time (Figure 1B).

**Table 1:** Baseline characteristics of the 584 participants by bedtime.

	**At or before 9 p.m**.	**9–11 p.m**.	**After 11 p.m**.	***P*-value**
*N* (%)	238 (22.8%)	675 (64.5%)	133 (12.7%)	
Age	75.6 (SD 8.0)	70.7 (SD 7.9)	68.8 (SD 7.5)	0.71
Female	139 (58.7%)	392 (58.3)	64 (48.1%)	0.08
Income≥1200RMB/month	231 (97.0%)	663 (98.2%)	128 (96.1%)	0.380
College education or more	32 (13.5%)	234 (34.8%)	53 (39.9%)	**<0.001**
BMI	25.5 (SD 3.8)	24.9 (SD 3.3)	24.7 (3.6)	0.186
Current smoking	14 (5.9%)	54 (8.1%)	26 (19.6%)	**<0.001**
Alcohol drinking	23 (9.8%)	44 (6.6%)	16 (12.0%)	**0.05**
Physical activity	83 (35.6%)	202 (30.4%)	54 (40.9%)	**0.04**
Depression symptoms	31 (12.9%)	84 (12.4%)	17 (12.6%)	0.990
Hypertension	161 (67.9%)	368 (54.7%)	68 (51.1%)	**0.001**
Heart disease	44 (18.6%)	97 (14.5%)	17 (12.9%)	0.23
Diabetes	49 (20.7%)	113 (16.8%)	12 (9.0%)	**0.02**
APOE e4	45 (21.6%)	89 (14.4%)	19 (14.6%)	**0.04**
Sleep duration	6.9 (SD 1.3)	6.9 (SD 1.2)	6.7 (SD 1.1)	0.245
Sleep efficiency	85.0 (SD 13.6)	83.1 (SD 14.4)	85.7 (SD 12.6)	0.171
PSQI score	4.48 (SD 3.07)	4.87 (SD 3.45)	4.91 (SD 3.14)	0.538
MMSE	27.6 (SD 2.9)	28.1 (SD 2.2)	28.6 (SD 2.0)	**0.01**
MMSE at follow-up^*^	23.8 (SD 7.1)	27.3 (SD 2.9)	27.4 (SD 3.8)	**<0.001**
Incident of dementia^*^	26 (24.5%)	20 (5.0%)	1 (1.2%)	**<0.001**

* *Among the 584 participants who completed the follow-up interview. Bold values indicate statistically significant*.

Table 2 indicates the HR of incident dementia by bedtime and rise time reporting from the participants. Unadjusted HRs and **those from** model 1 were 5.31 (95%CI 2.96–9.51) and 2.72 (95% CI 1.50–4.96). After adjustment for demographics, education, income, BMI, depressive symptoms, smoking, alcohol use, physical activity, comorbidities, APOE4 genotype, and baseline MMSE, those with a bedtime of before 9 p.m. were almost three times more likely to develop dementia [HR=2.16 (95%CI: 1.06–4.40)], compared to those going to bed between 9 and 11 p.m. Later bedtime (i.e., after 11 p.m.) showed the opposite but had a non-significant association with dementia risk (unadjusted HR = 0.24, 95%CI 0.03–1.80; adjusted HR in model 1=0.24, 95% CI 0.03–1.81; adjusted HR in model 2 = 0.15, 95%CI: 0.02–1.29). The association slightly attenuated (adjusted HRs in model 3 were 2.00 (95%CI: 0.94–4.29) for earlier bedtime and 0.12 (95%CI: 0.01–1.05) for later bedtime) after further adjustment for sleep duration and efficiency. We did not find an association for rise time and risk of dementia.

**Table 2:** Hazard ratio (95%CI) of dementia by bedtime and rise time.

**Bedtime**	**At or before 9 p.m**.	**9–11 p.m**.	**After 11 p.m**.
Incident of dementia, *N* (%)	26 (24.5%)	20 (5.0%)	1 (1.2%)
Unadjusted HR	5.31 (2.96–9.51)	1	0.24 (0.03–1.80)
HR adjusted in model 1	2.72 (1.50–4.96)	1	0.24 (0.03–1.81)
HR adjusted in model 2	2.16 (1.06–4.40)	1	0.15 (0.02–1.29)
HR adjusted in model 3	2.00 (0.94–4.29)	1	0.12 (0.01–1.05)
**Rise time**	**At or before 6 a.m**.	**6–8 a.m**.	**After 8 a.m**.
Incident of dementia, *N* (%)	26 (9.1%)	21 (7.6%)	0
Unadjusted HR	1.25 (0.71–2.22)	1	-
HR adjusted in model 1	1.17 (0.65–2.09)	1	-
HR adjusted in model 2	1.49 (0.72–3.10)	1	-
HR adjusted in model 3	2.10 (0.93-4.74)	1	-

*Model 1: adjusted for age, sex, education*.

*Model 2: adjusted for age, sex, education, income, body mass index (BMI), depressive symptoms, smoking, alcohol use, physical activity, comorbidities, APOE4 genotype, and baseline MMSE*.

*Model 3: adjusted for age, sex, education, income, body mass index (BMI), depressive symptoms, smoking, alcohol use, physical activity, comorbidities, APOE4 genotype, baseline MMSE, sleep duration, and efficiency*.

## Discussion

We found that earlier bedtime, not rise time, was associated with an increased risk of dementia in older Chinese adults without dementia and this was independent of demographics, life style, comorbidities, and APOE genotype.

Our results suggest that earlier bedtime may be an early marker of AD through the prospective study design. This speculation is supported by a recent Mendelian randomization (MR) analysis from the largest genome-wide association studies of the UK Biobank (N = 446,118), the Psychiatric Genomics Consortium (N = 18,759), and the International Genomics of Alzheimer's Project (N = 63,926). This MR analysis found that higher risk of AD, based on genetic risk score, was significantly associated with being a “morning person,” who prefers going to bed and waking earlier and is less active in the first half of the night (15). Other studies have reported the association between sleep timing and risk of cognitive impairment. An Italian study of 48 patients with AD and age-matched non-dementia controls showed an advanced bedtime in AD patients, especially for moderate to severe cases (16). Another study compared melatonin levels and sleep onset in 30 older patients with mild cognitive impairment (MCI) with 28 healthy controls, and found that MCI patients had early melatonin secretion onset, but the melatonin levels did not differ between groups. Additionally, patients with MCI had greater wake after sleep onset and increased rapid eye movement sleep latency (17). An analysis with 11,247 older individuals from the Swedish Twin Registry raised evidence that delayed rising time predicted dementia incidence after the 17-year-follow up (18). Different with this study, we did not find an association of rising time with dementia onset risk. It might be induced by the limited sample size with the relatively shorter follow-up time.

The exact mechanism which can explain the association of sleep timing with dementia risk is still under investigation. The circadian clock regulates the timing of sleep. Circadian rhythms are generated in specific brain structures to control a complex network of coupled self-sustained clocks in the brain and in the peripheral organs (2). Mutations in the core circadian clock genes in mice and humans manifest as abnormal sleep patterns, including short sleep time, early or late sleep phase, or unstable and fragmented sleep-wake rhythms (19, 20). Age-related changes in sleep-wake cycles may cause circadian dysfunction and result in earlier bedtimes and waking times, increased sleep fragmentation, and increased daytime sleepiness, which might be early indicators of declining health in late life (21, 22). Notably, the association between sleep timing and risk of dementia was slightly attenuated after adjustment for sleep duration and efficiency. The association between circadian rhythms and dementia could be explained by other potential mechanisms, such as protein aggregation from the brain, inflammation, synaptic homoeostasis, and oxidative stress, which are key pathogenic processes in the development of neurodegenerative diseases (2).

Strengths of the current study include a well-representative community-dwelling sample of the Chinese elderly, prospective follow-up for a relatively long period of time, detailed examination of cognitive function, and adjudicated diagnosis of dementia by experts. Our study cannot avoid the following limitations. First, sleep timing and quality were collected by the self-reported PSQI questionnaire, which could include recall bias. Second, even though we followed the participants for an of average 5 years, it may not be long enough to distinguish risk of dementia vs. an early marker of dementia. A longer follow-up can provide a solution for such reverse causality. Third, the relatively high lost-to-follow up rate with fewer incident dementia cases does not allow us to analyze the association with dementia subtypes. Fourth, there are still some potential confounding factors which could not be collected, but may impact the sleep-dementia association, although we tried to adjust covariables as much as possible in our multivariable statistical models.

## Conclusion

Our findings demonstrate that earlier sleep timing in older adults without dementia was associated with an increased risk of dementia. Future studies should examine the underlying mechanisms of this association and explore the usefulness of sleep timing as a preclinical marker for dementia.

## Data Availability Statement

The raw data supporting the conclusions of this article will be made available by the authors, without undue reservation.

## Ethics Statement

The studies involving human participants were reviewed and approved by Medical Ethics Committee of Huashan Hospital, Fudan University, Shanghai, China. The patients/participants provided their written informed consent to participate in this study.

## Author Contributions

XL, DD, and YL contributed to the design of the study, data analysis and interpretation, and drafting of the manuscript. All authors contributed to critical revision of the manuscript for important intellectual content.

## Conflict of Interest

The authors declare that the research was conducted in the absence of any commercial or financial relationships that could be construed as a potential conflict of interest.
